# Phenotype, genotype of neonatal diabetes mellitus due to insulin gene mutation

**DOI:** 10.1186/1687-9856-2015-S1-P12

**Published:** 2015-04-28

**Authors:** Can Thi Bich Ngoc, Vu Chi Dung, Bui Phuong Thao, Nguyen Ngoc Khanh, Nguyen Phu Dat, Maria Craig, Sian Ellard, Nguyen Thi Hoan

**Affiliations:** 1 Department of Endocrinology, Metabolism and Genetics. Vietnam National Hospital of Paediatrics, Hanoi, Vietnam; 2 Hanoi Medical University, Hanoi, Vietnam; 3 The Children Hospital at Westmead, Sydney, NSW, Australia; 4 Molecular Genetics, Old Path Lab, Royal Devon & Exeter Hospital, Barrack Road, Exeter, UK

## 

Insulin (INS) gene mutations that cause permanent neonatal diabetes mellitus change single protein building blocks (amino acids) in the protein sequence. These mutations are believed to disrupt the cleavage of the proinsulin chain or the binding of the A and B chains to form insulin, leading to impaired blood sugar control. At least 10 mutations in the INS gene have been identified in people with permanent neonatal diabetes mellitus.

## Objective

To describe clinical features and laboratory manifestations of patients with INS gene mutation and to evaluate outcome of management.

## Subject and methods

Clinical features, biochemical finding, mutation analysis and management outcome of 3 cases from 3 unrelated families were studied. All exons of INS gene were amplified from genomic DNA and directly sequenced.

## Results

3 cases (one girl and two boys) onset at 126.6 ±56.7 days of age with gestation age of 38.0 ± 1.4 weeks, birth weight of 2850 ± 494.9 g. All of them admitted with the feature of diabetic ketoacidosis with pH of 6.94 ±0.16; HCO^-^_3_ 2.63 ±0.85 mmol/l; BE 26.05±4.03 mmol/l, plasma glucose levels were 37.57±15.2 mmol/l, HbA1C of 9.9 ±2.5%. Mutation analysis of the INS gene showed: heterozygous for a novel missense mutation (c.127T>A; C43S) in exon 2 of INS gene in one case; heterozygous for a novel INS splicing mutation, c.188-31G>A of the INS gene in two cases. After 8 months of insulin treatment, two patients with c.188-31G>A mutation have normal development with DQ 80-100%, HbA1C of 6.85 ±0.49%, quite normal blood glucose levels. The case with c.127T>A mutation treated with insulin for 8 years has physical development delay, poor blood glucose control with HbA1C of 11.4%.

## Conclusions

It is important to perform screening gene mutation for patients with diabetes diagnosed before 6 months of age to control blood glucose and follow up the patients.

